# Improving the Diagnosis, Management, and Outcomes of Children with Pneumonia: Where are the Gaps?

**DOI:** 10.3389/fped.2013.00029

**Published:** 2013-10-23

**Authors:** Anne B. Chang, Mong H. Ooi, David Perera, Keith Grimwood

**Affiliations:** ^1^Queensland Children’s Respiratory Centre, Queensland Children’s Medical Research Institute, Queensland University of Technology, Brisbane, QLD, Australia; ^2^Child Health Division, Menzies School of Health Research, Charles Darwin University, Darwin, NT, Australia; ^3^Department of Pediatrics, Kuching Hospital, Sarawak, Malaysia; ^4^Institute of Health and Community Medicine, Universiti Malaysia Sarawak, Kota Samarahan, Malaysia; ^5^Queensland Children’s Medical Research Institute, The University of Queensland, Brisbane, QLD, Australia; ^6^Queensland Paediatric Infectious Diseases Laboratory, Royal Children’s Hospital, Brisbane, QLD, Australia

**Keywords:** pneumonia, acute respiratory infections, diagnosis, treatment, outcomes

## Abstract

Pneumonia is the greatest contributor to childhood mortality and morbidity in resource-poor regions, while in high-income countries it is one of the most common reasons for clinic attendance and hospitalization in this age group. Furthermore, pneumonia in children increases the risk of developing chronic pulmonary disorders in later adult life. While substantial advances in managing childhood pneumonia have been made, many issues remain, some of which are highlighted in this perspective. Multiple studies are required as many factors that influence outcomes, such as etiology, patient characteristics, and prevention strategies can vary between and within countries and regions. Also, outside of vaccine studies, most randomized controlled trials (RCTs) on pneumonia have been based in resource-poor countries where the primary aim is usually prevention of mortality. Few RCTs have focused on medium to long-term outcomes or prevention. We propose different tiers of primary outcomes, where in resource-rich countries medium to long-term sequelae should also be included and not just the length of hospitalization and readmission rates.

## Why Childhood Pneumonia is Important

Childhood pneumonia is of worldwide importance for several reasons. Firstly, while many illnesses receive relatively more attention, pneumonia remains the most important cause of mortality and morbidity in young children globally (1, 2). Secondly, deaths from pneumonia are largely preventable in this age group. Thirdly, pneumonia, especially when recurrent, is linked to future chronic lung disease (3). Thus, interventions that reduce pneumonia and acute lower respiratory infections (ALRIs) have both short and long-term benefits. This perspective is not a comprehensive review of childhood pneumonia and its management. Instead, it aims to highlight the many gaps in our knowledge so as to stimulate and improve clinical research in pneumonia that will lead subsequently to improved clinical care.

## Global Pneumonia Burden

Pneumonia is the largest (18%) single cause of death in children aged <5-years (1). Global estimates of the annual incidence of pneumonia in children aged <5-years range from 120 to 160 million episodes, with more than 99% occurring in resource-limited countries (1). While there are substantial inter-country and inter-continental differences in the annual incidence of pneumonia [0.33 episodes per child-year in Africa, 0.05 in developed countries (1, 2)], there is also intra-continental (4) and wide intra-country variability (1, 5). For example, in South America, the percentage of childhood deaths under the age of 5-years attributable to pneumonia is much lower in Chile and Uruguay (5–10%) than in Bolivia, Peru, and Guyana (15–20%) (4). Also, in contrast to the rest of affluent Australia, ALRIs (encompassing pneumonia) are the commonest cause of preventable deaths in infants, emergency medical retrievals from remote communities, and hospitalizations among Indigenous children aged <5-years (6, 7). Hospitalized-pneumonia incidence of infants in the Northern Territory of Australia (the region with the highest proportion of Indigenous people) is 0.43 per child-year (8). Similarly, Indigenous children in New Zealand and the United States (US) also bear a substantially disproportionate burden of disease (9, 10). Nevertheless, while there is little doubt that socio-economic issues are important, pneumonia remains one of the most common causes of hospitalization in children, even in resource-rich countries where before the widespread adoption of pneumococcal conjugate vaccines (PCVs) an estimated 1.5 million children aged <5-years were admitted annually.

### The knowledge gaps

Epidemiological estimates of pneumonia depend upon the accuracy of data collection, which is problematic because of: (a) absence of a diagnostic gold standard, (b) lack of resources to collect data systematically (especially in resource-poor countries), and (c) considerable intra-country variation making extrapolation of data subject to biases, especially when universal health systems are sub-optimal and people seek treatment in the private sector (where data capture is more difficult).

## Etiology

Acute lower respiratory infections are caused by several respiratory viral and bacterial pathogens, often in combination with one another. Major viral causes of ALRI and pneumonia in infants and children include respiratory syncytial virus, influenza, adenoviruses, parainfluenza 3, and human metapneumovirus. While viruses are the predominant cause of ALRIs, especially in the young, respiratory bacterial pathogens are most often implicated in childhood deaths from pneumonia (1). The predominant bacterial pathogen is *Streptococcus pneumoniae*, which dominates across all age groups, but other important pathogens include *Haemophilus influenzae, Staphylococcus aureus* and, in older children, *Mycoplasma pneumoniae* (11). Other common causes of severe pneumonia are measles in regions where vaccine uptake is poor, non-typhoid *Salmonella* species in regions of Africa where malaria is endemic, *Klebsiella pneumoniae* in malnourished children and neonates, and *Pneumocystis jirovecii* and *Mycobacterium tuberculosis*, especially in HIV positive infants and children.

Current studies determining the etiology of pneumonia vary substantially with respect to factors that influence the diagnosis and ascribed microbial etiology. These include case definitions, use of and interpretation of chest x-rays (CXR), peripheral blood white cell counts, and inflammatory markers, depth of investigations, facility type, and patient characteristics. Consequently, studies describe different frequency and types of pathogens associated with pneumonia. The Pneumonia Etiology Research for Child Health (PERCH) project is the largest multisite study (6000 children, 7 African and Asian countries) of childhood pneumonia. It seeks to address the aforementioned limitations by using case-control methodologies and adopting a protocol that has standardized enrollment criteria, specimen collection, laboratory testing, and molecular detection techniques (12).

### The knowledge gaps

Despite advances in identifying microorganisms using highly sensitive molecular techniques, ascribing causation is problematic. Nucleic acid amplification (NAA) techniques, such as polymerase chain reaction (PCR) assays, identify genetic material, but the implicated microbe may no longer be viable or infectious and their presence may be from a recent, but unrelated respiratory illness. For example, the prevalence of respiratory viruses detected by molecular techniques in asymptomatic children is as high as 42% (13) and strains of adenovirus C may remain latent in mucosal lymphocytes and be shed for months or even years (14).

Even when the same molecular detection techniques for viruses are used, the site of specimen collection influences results. In paired comparisons of concurrently obtained upper and lower airway specimens for respiratory viruses in 75 children, we found significant discordance between nasopharyngeal aspirate and bronchoalveolar lavage (BAL) specimens (manuscript submitted) (15). The discordance was dependent on the virus type and most marked for human rhinovirus and adenoviruses. Additionally, we (16) and others (17) have found that even when investigating viral infections from a single specimen collection site, detection of multiple viral types is not uncommon. This observation compounds the difficulty in determining the primary causative agent and presents new questions about the roles of these viruses in the etiology of the disease.

The ideal samples for determining etiologic agents in bacterial pneumonia are lower airway specimens. It is usually neither necessary nor feasible to obtain either BAL or needle lung aspirate specimens in acute pneumonia. Induced sputum is an alternative only in older children, and as potential respiratory bacterial pathogens commonly colonize the upper airways of healthy children, oropharyngeal contamination can complicate interpretation of culture results. Blood cultures are also infrequently (<10%) positive in children with pneumonia and as PCVs become incorporated into national immunization schedules their sensitivity is likely to be further reduced (18). PCR techniques have only modestly increased the yield of pathogen detection in blood samples (19), while with the exception of *M. pneumoniae*, serology is impractical in most clinical situations. Thus, it remains a challenge to determine the ideal, yet feasible, specimen for identifying the etiological agent(s) in pneumonia.

Increasingly, viral–viral, viral-bacterial, and bacterial–bacterial interactions in the pathogenesis of respiratory infections are recognized with *in vitro* and *in vivo* animal (20) and human studies (21, 22). Thus, although viruses may initiate the respiratory infection, secondary bacterial infection may occur, and simply identifying a virus at presentation (leading to antibiotics being with-held) may not indicate the sole etiology of the child’s acute clinical presentation or determine its long-term outcomes.

The complexity of the microbial contribution is further increased by introducing the world of “-omics” (e.g., metagenomics). While interest in this field is exploding, its use may further complicate ascribing etiology to a single organism. Although analysis of gene expression signatures shows considerable promise for identifying causative pathogens of pneumonia (23), these and other NAA platforms (e.g., microbead arrays, microarray) are unlikely to be made available in the near future to resource-poor countries where the burden of childhood pneumonia is greatest.

## Prevention and Contributing Factors

Vaccines, such as pertussis, measles, and more recently Hib and PCVs, have reduced the worldwide incidence of pneumonia (24). However, the benefit from population vaccination programs is not always uniform, as for example in the case of PCV-7, which although having significantly reduced pneumonia rates in target and some older age groups (24), its impact on other populations living in resource-rich nations has been surprisingly limited. Despite achieving high population PCV-7 vaccination rates of ∼90%, the incidence of World Health Organization (WHO)-defined radiographic pneumonia was not reduced in the Indigenous population living in the Northern Territory of Australia (25).

In resource-poor nations, important factors identified for increasing the risk of pneumonia include overcrowding, access to clean water, malnutrition, anemia, young maternal age, low birth weight, and exposure to tobacco smoke and other environmental pollutants. Several of these risk factors also contribute to children in resource-rich nations developing pneumonia.

### The knowledge gaps

The contribution of asthma or asthma-like illness to pneumonia remains controversial (26). While people with asthma have minor intrinsic abnormalities in their immune responses (27) leading to a predisposition to viral lung infections and occasionally invasive pneumococcal disease, it is also possible that some children with asthma may have been misdiagnosed as pneumonia (26). Further information on the contribution (e.g., misdiagnosis or co-morbidity) of the asthma phenotype to early childhood pneumonia, especially in resource-limited countries, is warranted (26).

Good hygiene practices reduce ALRIs and promotion of safe hygiene is the single most cost-effective means of preventing infectious disease (28). However, how best to make safe hygiene practices a matter of daily routine is yet to be determined (28).

In some settings, such as in Indigenous settings in the US and Australia, a combination of factors underlie why the young children living in remote communities experience a high burden of pneumonia. While housing is very important, housing upgrades can be insufficient to improve health outcomes in children and a broader approach is required (29). Exactly what this entails is yet to be determined, but factors likely to be important are adequate water supply and minimizing indoor air pollution.

## Diagnosis

Current case definitions for pneumonia vary considerably and are highly setting dependent (18). In WHO-defined pneumonia, cough or breathing difficulties, and age-adjusted tachypnea are sufficient for diagnosing mild-to-moderate pneumonia, but these criteria were designed for health workers with relatively little training. However, the diagnostic thresholds for respiratory rate differ between the WHO, US, and United Kingdom (UK) guidelines (30, 31). Furthermore, even within affluent countries such as the US, the diagnostic criteria, and testing for pneumonia within hospital Emergency Departments varies widely (32).

Community healthcare workers often diagnose pneumonia on history and examination (respiratory rate, dyspnea, auscultatory findings). However, in young children with a cough, chest auscultatory findings, even by doctors (general practitioners), are unreliable with kappa values (0.39, 95% CI 0.26–0.53) (33) below the acceptable clinical range.

While many studies use CXRs as the gold standard (18), there is disagreement over whether a CXR should be an index test. Current USA (31) and UK (34) guidelines on childhood pneumonia do not advocate CXRs outside of hospital settings. Furthermore, their interpretation is subjective often resulting in additional diagnostic variability. Also, CXRs are insensitive when compared to chest computed tomography (CT) scans (35). While chest CT scans will not be used to diagnose pneumonia in the usual clinical setting, the poor sensitivity of CXRs means they too cannot be used as a diagnostic gold standard.

Laboratory tests (e.g., C-reactive protein, peripheral blood white cell count, erythrocyte sedimentation rate) are ancillary and non-diagnostic tests. In adults with community-acquired pneumonia, employing procalcitonin levels to initiate and cease antibiotics might be useful, however, procalcitonin diagnostic thresholds in childhood pneumonia are less defined and their usefulness and safety in guiding management has not been established (18).

### The knowledge gaps

World Health Organization has published a standardized method to define radiographic pneumonia (based on CXRs) for epidemiological studies and vaccine trials (36). The WHO definition for primary endpoint consolidation WHO-EPC (36) was framed to be more specific for likely bacterial pneumonia than those used for clinical purposes. However, some authors have applied the WHO-EPC definition in the clinical context (24), an approach that we do not recommend (37). In a blinded study, a pediatric pulmonologist’s assessment of radiographic pneumonia in a cohort of children hospitalized with clinical pneumonia, had significantly higher positive predicted value for the presence of auscultatory crackles and elevated peripheral blood white cell counts when compared to WHO-EPC read by a panel standardized to interpret WHO-EPC (37). Others have raised similar concerns, with left lower and right middle lobe consolidation and non-alveolar pneumonia causing the greatest levels of disagreement (37, 38).

A systematic review compared 11 different “gold standards” for identifying bacterial pneumonia and found that diagnostic tests used for pediatric pneumonia have not been validated rigorously (18). Some advocate chest ultrasounds for diagnosing pneumonia (24), but this approach has not been validated. While chest ultrasounds may provide adequate images of some lobes, they will not for others, particularly the right middle lobe. Thus, using another sub-optimal diagnostic tool will only complicate the field further.

The lack of a universally agreed diagnostic gold standard for childhood pneumonia, especially one that can also differentiate between bacterial and non-bacterial pneumonia is a major limitation in clinical research in this area. Vaccine probes have proven useful at a population level, but they under-estimate the burden of disease as no vaccine is 100% efficacious or, in the case of PCVs, protects against all disease causing serotypes. Instead, valid standards useful for various scenarios should be developed. These include standards for clinical, epidemiological, and treatment purposes, such as defining when antibiotics should be used (as opposed to conceptually a purely bacterial or viral-based etiology).

## Management

Managing childhood pneumonia relies upon several factors:

- Child factors
  - Demographics (e.g., age, immunization status, household contacts, etc.)
  - Severity of pneumonia (e.g., hypoxemia, complications)
  - Co-morbidities (e.g., malnutrition, HIV, other immunodeficiencies, malaria, underlying cardiac, pulmonary, neuromuscular, or metabolic disorders)
- External factors
  - Country (health care facilities)
  - Setting (urban vs. rural; hospital vs. community)

Pneumonia case management, especially in resource-poor regions, is important (39). These focus on: (a) antimicrobial therapy when appropriate, (b) correcting hypoxemia, (c) fluid and nutritional management, (d) treatment of co-morbidities, and (e) close observation for developing complications. National treatment guidelines exist (31, 34), but there is wide variability in managing pneumonia, even in resource-rich countries (40).

### The knowledge gaps

Some issues specific to pneumonia management in resource-poor settings were highlighted recently (39). These included difficulties in differentiating clinically between pneumonia and other common serious illnesses in infants and young children living in these regions, such as bronchiolitis and malaria, issues over when to refer for inpatient management, antibiotic choice when HIV, tuberculosis, and *Salmonella* infections are common, micronutrients use, definition of hypoxemia, and defining and managing treatment failure in the acute setting.

While basic management principles are known, the details of the “how, when and what” remain uncertain. For example, with oxygen; when should it be initiated, what pulse oxygen saturation should be targeted once oxygen supplementation is commenced and how best to deliver oxygen (pure or mixed, high or low flow) is still unknown. Because of the cost implications for resource-poor countries and randomized controlled trial (RCT) evidence that high concentration oxygen therapy may result in adverse outcomes in adults with acute asthma (41), these issues that might seem unimportant, are highly relevant in improving clinical care.

The many RCTs involving antibiotics have so far only focused on short-term outcomes and increasingly shorter courses of antibiotics have been advocated. None however, have focused on the length of wet or productive cough post-treatment. As with oxygen, while this may appear unimportant, the persistence of chronic wet cough (signifying persistence of excessive airway secretions and lower airway infection) is associated with developing bronchiectasis in children (42).

Further, outside of vaccine trials, most RCTs on treating pneumonia in the last two decades were based in resource-poor areas (30) where risk factors, etiology, patient characteristics, and settings differ substantially from resource-rich countries. Meta-analysis of studies have shown discrepancies in results between studies conducted in resource-poor vs. resource-rich settings (43). Those conducted in resource-poor regions have in some cases had significantly more favorable treatment effects than in resource-rich settings (43).

The primary objective of childhood pneumonia management has focused largely on preventing mortality in resource-poor countries and reducing hospitalization rates in resource-rich nations. Here, we propose three tiers for managing pneumonia in children. With the increasing awareness of chronic disease in developing countries (including lung disease) and that a substantial amount of chronic lung disease originates in childhood (42, 44, 45), in an ideal world all tiers should be targeted at clinical practice. The main objectives of each tier and on-going research questions within each of these objectives include:

- Preventing mortality
  Identifying who needs hospitalization
  Recognizing who needs oxygen
  When to start antibiotics and in whom and for how long?
- Reducing short-term morbidity
  Type and duration of antibiotic courses
  Duration of oxygen supplementation and weaning
  Identifying and treating co-morbidities, including malnutrition
  Preventing and managing complications
- Promoting return to full and long-term health
  Identifying and treating on-going symptoms post-acute episode
  Preventing recurrences and long-term sequelae

## Consequences of Pneumonia

### Acute phase

Simple pneumonia may evolve into complicated pneumonia, defined by developing parapneumonic effusions, empyema, pyopneumothorax, or necrotizing pneumonia. Interestingly, the incidence of complicated pneumonia has increased over the last two decades and has occurred in countries before and after introducing PCVs into the immunization schedule (46), although there are encouraging signs from the UK that the incidence may now be declining after changing from a PCV-7 to PCV-13 schedule. However, it is beyond the scope of this article to discuss complicated pneumonia further.

### Short-term

Few studies have closely followed-up children with pneumonia. In a study of 78 Australian Indigenous children hospitalized with pneumonia and followed for 12-months afterward, 26% had new treatable chronic respiratory symptoms identified (47). Meanwhile, in a New Zealand study, 74% of the 81 children with an adequately performed CXR 10–14 months post-hospitalization for an ALRI had features of on-going respiratory morbidity (wet cough, auscultatory chest crackles, CXR abnormalities) (5).

### Long-term

The alveolar stage of human lung development occurs from 36-weeks gestation and continues for at least 7-years post-natally (48). Hence, early infectious or inflammatory insults in the first few years of life, when postnatal lung development is the most important, are most likely to result in long-term effects. While low birth weight can influence future lung function, there is increasing evidence that early life events, such as pneumonia, are at least equally important determinants of adult lung dysfunction, as shown in human and animal studies (44, 45, 49, 50).

In adult cohort studies, recurrent ALRIs are independent risk factors for subsequent chronic obstructive pulmonary disease (44, 49), although they did not confirm the history of prior childhood pneumonia. There are very few long-term pediatric follow-up studies. One case-control study on bronchiectasis showed that hospitalized pneumonia was a risk factor, particularly when it was recurrent and/or severe (51). An English study of 103 children found that children post (median 5.6-years) hospitalized pneumonia were significantly more likely to have persistent cough or asthma than controls (52). Similarly, a Gambian-based study showed that 68 children hospitalized previously with pneumonia had significantly higher odds (2.8, 95% CI 1.1–7.4) of chronic lung disease at follow-up 12–14 years later (53).

### The knowledge gap

Many gaps in persistent morbidity and long-term sequelae from pneumonia exist. The concept of the post-bronchiolitis syndrome is appreciated increasingly (54) and in the last decade the WHO has emphasized the origins of adult chronic lung disease from early in life and that potential intervention trials should begin during this critical developmental stage of lung growth and development.

## Summary

We have highlighted some of the key knowledge gaps in diagnosing and managing childhood pneumonia. Clinical trials are now needed in various settings to address these gaps. We propose a tiered approach and a framework to advance this field so as to reduce the short and long-term burden associated with pneumonia in children.

## Authors Contribution

Anne B. Chang conceptualized the manuscript and wrote the first draft. Keith Grimwood, Mong H. Ooi, and David Perera amended the manuscript.

## Conflict of Interest Statement

Anne B. Chang has received funding from GlaxoSmithKline for an investigator driven study on the effects of Synflorix^®^ on airway bacteriology. Keith Grimwood has participated on advisory boards to GlaxoSmithKline on pneumonia and pneumococcal conjugate vaccines. Mong H. Ooi and David Perera have no conflict of interest.
